# Diagnostic Value of Karyotype, Microarray, RASopathy Gene Testing and Ultrasound in Fetuses with Nuchal Translucency 3.0–3.4 mm: A Single-Center Cohort Retrospective Study

**DOI:** 10.3390/genes17020234

**Published:** 2026-02-12

**Authors:** Silvia Andrietti, Giuseppe Gullo, Diliana Beleva, Alessia Maccarrone, Lina De Paola, Chiara Roberta Gaggero, Chiara Calcagno, Maria Lucia Furnari, Pierangela De Biasio

**Affiliations:** 1 Prenatal Diagnosis and Perinatal Medicine Unit, IRCCS Ospedale Policlinico San Martino, 16132 Genova, Italy; diliana.beleva@hsanmartino.it (D.B.); chiara.gaggero@hsanmartino.it (C.R.G.); chiara.calca@gmail.com (C.C.); pierangela.debiasio@hsanmartino.it (P.D.B.); 2 Department of Obstetrics and Gynecology, Villa Sofia Cervello Hospital, University of Palermo, 90146 Palermo, Italy; 3 Department of Neurology, Rehabilitation, Ophtalmology, Genetics, Maternal and Infant Health (DiNOGMI), 16132 Genoa, Italy; alessia.maccarrone97@gmail.com; 4 Department of Anatomical, Histological, Forensic and Orthopedic Sciences, Sapienza University of Rome, 00161 Rome, Italy; lina.depaola@uniroma1.it; 5 AOOR Villa Sofia-Cervello, 90146 Palermo, Italy; marialucia.furnari@villasofia.it

**Keywords:** nuchal translucency, prenatal diagnosis, chromosomal abnormalities, chromosomal microarray, RASopathies, first-trimester screening

## Abstract

**Background**: Increased nuchal translucency (NT) is associated with an elevated risk of genetic abnormalities and structural malformations. The clinical utility of invasive testing and the optimal diagnostic approach in mildly increased NT (3.0–3.4 mm) is debated. This study aimed to evaluate genetic and ultrasound findings in this subgroup and to assess the diagnostic yield of advanced genetic testing. **Methods**: We retrospectively included a total of 107 fetuses with NT between 3.0 and 3.4 mm from a single fetal medicine unit. Complete outcome data were available for 97 pregnancies. Invasive prenatal testing with standard karyotype, chromosomal microarray analysis (CMA) and RASopathy panel testing were offered. All patients underwent detailed ultrasound examination to detect structural abnormalities at 16 and 20 weeks, regardless of whether invasive testing was performed. **Results**: Invasive prenatal testing, amniocentesis or chorionic villus sampling, (CVS), was performed in 77/97 cases (79.4%). Genetic abnormalities were detected in 28/97 (28.9%). Overall, five rare genetic anomalies were identified; none would have been detected by quantitative fluorescent polymerase chain reaction (QF-PCR) or non-invasive prenatal testing (NIPT). Two anomalies were detectable by standard karyotype, two exclusively by CMA and one exclusively by RASopathy panel. When considering all cases undergoing advanced genetic testing (CMA or RASopathy panel, *n* = 35) the overall diagnostic yield was 8.5% (3/35). When calculated across the entire cohort with complete follow-up, the additional diagnostic yield was 3.1% (3/97). Major structural malformations were identified in 17/97 cases (17.5%), of which 10 (58.8%) were associated with genetic abnormalities. **Conclusions**: Fetuses with NT measurements between 3.0 and 3.4 mm show a substantially increased risk of genetic abnormalities and structural malformations. These findings support a comprehensive prenatal evaluation, including invasive testing with advanced genetic analysis and detailed ultrasound assessment, to optimize diagnosis and counseling.

## 1. Introduction

Measurement of nuchal translucency (NT) between 11 and 13 weeks of gestation is a pivotal element of first-trimester aneuploidy screening. Increased NT is defined as a thickness greater than the 99th centile or 3.5 mm in the first trimester, irrespective of gestational age [1]. An increased NT is not only a strong marker for chromosomal abnormalities, but also indicates a higher risk of major congenital malformations, genetic syndromes—including RASopathies—and adverse perinatal outcomes, underscoring its critical role in early fetal assessment [2,3,4,5,6,7]. Clinical attention has focused on NT values above 3.5 mm, which are universally recognized as an indication for invasive prenatal testing [1]. However, the optimal management of fetuses with NT between 3.0 and 3.4 mm remains controversial [8,9].

Moreover, in recent years, non-invasive prenatal testing (NIPT) has increasingly been adopted as a first-line screening tool for common fetal aneuploidies (particularly trisomies 21, 18 and 13), even in the absence of a formal first-trimester combined risk assessment that incorporates NT measurement and serum markers [10]. Accumulating evidence suggests that NT values in the range of 3.0 to 3.4 mm also carry relevant risk, even when the combined first-trimester screening indicates low probability for aneuploidy. Several studies demonstrate that fetuses within this NT range may still harbor copy-number variants (CNVs), single-genes disorders, or structural malformations identifiable later in pregnancy. A subtle increase in NT thickness can also be an early marker of impaired lymphatic development, cardiac dysfunction, or altered extracellular matrix signaling, as seen in RASopathies [11,12,13]. Therefore, in pregnancies where the NT is mildly increased (e.g., 3.0–3.4 mm), a “low risk” NIPT result for trisomies 21, 18 and 13 does not exclude a significant residual risk of rare CNVs, single-gene disorders, balanced rearrangements, or many other structural genomic abnormalities [14,15,16,17,18,19,20,21].

In this context, recent large-scale studies have begun to explore the diagnostic yield of advanced genetic testing across different NT thresholds. Ji et al. reported a cohort of 2328 pregnancies with increased NT, demonstrating a clear correlation between NT thickness and the prevalence of genetic anomalies [22]. In particular, even among fetuses with NT measurements between 3 and 3.4 mm, chromosomal microarray analysis (CMA) identified pathogenic variants not detectable by conventional karyotyping, while the addition of whole genome sequencing further increased diagnostic yields, even if less markedly than in cases with higher measurements.

Despite these observations, current international guidelines differ substantially in their recommendations for this intermediate NT range. Clinical management of these fetuses remains heterogeneous across centers: some protocols recommend expectant management with detailed second-trimester anatomy scans, while other advocate offering invasive prenatal testing and chromosomal microarray, regardless of screening.

The American College of Obstetricians and Gynecologists recommends considering invasive testing at NT ≥ 3.0 mm [10], while other scientific societies such as the International Society of Ultrasound in Obstetrics and Gynecology (ISUOG) maintain the 3.5 mm threshold [1]. Moreover, most prenatal series and expert groups suggest that RASopathy testing should be considered after CMA results are normal when NT measures more than 5 mm in isolated cases or when NT is ≥3.5 mm in the presence of additional anomalies [1]. However, there is a notable gap in the literature for fetuses with NT measurements between 3.0 and 3.4 mm. This intermediate group is underrepresented in published studies, and few data are available to clarify the diagnostic yield or clinical utility of RASopathy testing in this specific NT range. As a result, recommendations for this subgroup remain less evidence-based and are often extrapolated from cohorts with higher NT measurements. The divergence in guidelines reflects ongoing uncertainty regarding the magnitude of risk in fetuses with NT measurements in the 3.0–3.4 mm range, and whether this risk is sufficient to justify invasive testing. This uncertainty is further compounded by the need to balance the potential diagnostic benefit against procedure-related risks, as well as the advantages and limitations of complex genetic analyses, including issues of variant interpretation, turnaround time, and parental counseling.

Clarifying the diagnostic yield of different methods of genetic analysis and outcome spectrum in this specific NT range is essential for guiding counseling and decision-making. The present study aims to contribute to this discussion by analyzing a cohort of 107 pregnancies with NT 3.0–3.4 m, assessing genetic diagnostic yield in the subgroup undergoing invasive testing, prevalence of structural anomalies and pregnancy outcomes.

## 2. Methods

This is a retrospective cohort study of women carrying a fetus with an NT between 3.0 and 3.4 mm at 11 + 0–13 + 6 weeks of ultrasound performed in a single, tertiary center. As we are a referral center, some women were referred for evaluation after the finding of an NT between 3.0 and 3.4 mm. Data from 2016 to 2025 were included in the analysis.

NT measurements were performed by certified fetal medicine specialists, trained according to Fetal Medicine Foundation (FMF) criteria, with crown–rump length set as between 45 and 84 mm. Exclusion criteria included NT measurements outside the 3.0–3.4 mm range. Cases were identified through the electronic database (Excel version 15.0, Microsoft Corp, Redmond, WA, USA) using standardized NT measurement records from first-trimester screening examinations.

The first-trimester combined screening was offered to all patients in accordance with national health recommendations [23]. Risk assessment for fetal aneuploidies was performed using the FMF algorithm, incorporating maternal characteristics, serum-free β human chorionic gonadotropin (hCG), pregnancy-associated plasma protein A (PAPP-A), and fetal NT thickness [19,24]. The analysis of PAPP-A and free beta was performed using the PerkinElmer DELFIA Xpress system (PerkinElmer, Inc., Waltham, MA, USA). The data entry program, Astraia (Astraia Software GMBH, Munich, version 29.2.1, DB 18857) was used to record prenatal ultrasound findings, biochemical markers, and prenatal risk assessment.

NT measurements were obtained in the midsagittal plane following FMF standardized techniques. Quality criteria included a neutral fetal position, magnification showing only the head and upper thorax, measurement of the widest point of translucency with calipers placed on the inner borders of the nuchal space, and clear distinction from the amnion [19,24].

Women with a risk ≥ 1:350 for trisomy 21 (T21), trisomy 13 (T13), or trisomy 18 (T18) were considered high-risk. According to local protocol, a fetal anatomy survey was performed, evaluating the fetal head and choroid plexus, upper and lower limbs, stomach, abdominal cord insertion, bladder, and the four-chamber heart view with color Doppler assessment including the V-sign [18,23].

All patients with NT of 3.0–3.4 mm received counseling from a fetal medicine specialist to discuss the implications of increased NT, including the risks of chromosomal and non-chromosomal abnormalities, structural defects, and available alternative screening methods.

In the study center, the management policy was to offer invasive testing in women whose fetuses had NT greater than 3 mm, high-risk result at the first-trimester screening, fetal anomalies or maternal age > 35 years old.

Diagnostic options, including chorionic villus sampling (CVS) and amniocentesis, were explained along with procedure-related risks. When couples opted for invasive testing, detailed information was provided regarding the available genetic tests, including the possibility of variants of uncertain significance (VUS). Invasive testing was performed by experienced operators under ultrasound guidance.

CVS was offered between 11 and 14 weeks of gestation, while amniocentesis was performed from 16 weeks onward. Selection of the procedure was guided by gestational age at presentation, maternal preference following counseling, and technical feasibility. When initial results were inconclusive, both procedures were performed to ensure diagnostic confirmation.

Genetic testing evolved during the study period as new technologies were adopted. All samples underwent standard G-banded karyotyping (≥20 metaphases). Quantitative fluorescence PCR (QF-PCR) was used for rapid aneuploidy detection of chromosomes 13, 18, 21, X and Y, with results typically within 24–48 h.

Since 2021, our local protocol has included CMA and RASopathy testing when NT is above 3 mm.

In most cases, CMA was performed using array comparative genomic hybridization (a-CGH), while in one case, a High-Density Single-Nucleotide Polymorphism array (HD-SNP array) was performed because the analysis was carried out in a laboratory using this platform. Genomic DNA was analyzed using the Human Genome CGH Microarray Kit 8x60K (Agilent Technologies, Santa Clara, CA, USA). Arrays were processed and scanned according to the manufacturers’ instructions. Data analysis was performed using Cytogenomics software version 5.2.0.20 (Agilent). Under optimal conditions, the array resolutions were approximately 100–150 kb in syndromic regions and 500 kb in the backbone regions. CNVs were identified based on a minimum of four consecutive probes showing significant imbalance.

The HD-SNP array was performed using a high-resolution whole-genome CytoScan™ 750K array (Affymetrix, Thermo Fisher Scientific, Santa Clara, CA, USA), with an analytical resolution for CNV detection of approximately 50–100 kb.

RASopathy testing was performed using next-generation sequencing panels targeting genes associated with Noonan syndrome, cardiofaciocutaneous syndrome, Costello syndrome, and related disorders. In silico gene panel analysis was performed focusing on the coding regions and exon–intron boundaries (±5 bp) of the following genes: PTPN11 (NM_002834), *SOS1* (NM_005633), *BRAF* (NM_004333), *RAF1* (NM_002880), *MAP2K1* (NM_002755), *MAP2K2* (NM_030662), *KRAS* (NM_004985.5), *NRAS* (NM_002524), *SHOC2* (NM_007373), *HRAS* (NM_005343), *RIT1* (NM_006912), *CBL* (NM_005188), *LZTR1* (NM_006767), *SOS2* (NM_006939), *PPP1CB* (NM_206876), *MRAS* (NM_001085049), *RRAS* (NM_006270), *RRAS2* (NM_012250), *SPRED2* (NM_181784), and *ERF* (NM_006494).

In one case, following genetic counseling, the NGS panel was expanded to include gene *CHD7* (NM_017780).

Next-generation sequencing (NGS) was performed in a trio-based (proband and both parents) approach using the KAPA Hyper Exome Probes V2 kit (Roche, Basel, Switzerland) on the NovaSeq 6000 platform (Illumina, San Diego, CA, USA). Sequencing achieved a mean depth of coverage > 100×, with >99% of targeted bases covered at ≥20×, ensuring robust detection of single-nucleotide variants (SNVs) and small insertions/deletions (indels) within the targeted regions. The analytical sensitivity and specificity of the assay were both greater than 99% for the reported variant classes.

Results were classified as pathogenic, benign, or VUS, according to ACMG guidelines [20].

All patients underwent detailed ultrasound surveillance, regardless of whether invasive testing was performed. The follow-up protocol included a targeted anatomical survey at 16 weeks, a standard second-trimester morphology scan at 20–22 weeks including fetal echocardiography performed by experienced fetal medicine specialists, a third-trimester growth assessment at 30–32 weeks, and additional scans as clinically indicated.

If an abnormal fetal karyotype and/or additional congenital defects were detected, follow-up ultrasound scans were scheduled according to the underlying fetal condition. The frequency and focus of these scans were tailored to monitor the specific anomalies identified, guide prenatal management, and to counsel the couple regarding prognosis and potential interventions.

Women were identified through the electronic database. Clinical characteristics, genetic finding and pregnancy outcomes were abstracted from the clinical records. For live-born infants in the cohort, follow-up was performed until hospital discharge after birth. No systematic postnatal follow-up data beyond this point were available.

Descriptive statistics were used to summarize the study population and outcomes, with variables presented as frequencies and percentages. Given the descriptive study design and limited sample size, formal hypothesis testing was generally not performed.

The detection rate of genetic abnormalities was calculated both among fetuses undergoing invasive testing (diagnostic yield) and in relation to the entire study cohort. This approach was adopted to distinguish between test performance and overall prevalence and to minimize selection bias.

## 3. Results

During the study period (November 2016 to September 2025), 107 pregnancies were identified with NT measurements between 3.0 and 3.4 mm at first-trimester screening.

Among these pregnancies, the majority (94/107, 87.8%) underwent first-trimester combined screening for major fetal aneuploidies. Of these, 9.6% (9/94) had a low-risk result and 90.4% (85/94) had a high-risk result. The remaining patients either proceeded directly to invasive testing (9.3%, 10/107) or declined further risk assessment (2.8%, 3/107). Following the initial NT finding, 9.3% (10/107) declined further follow-up and were lost to follow-up. The remaining 97 patients (90.6%) continued surveillance at our center. None of the patients who were lost to follow-up opted for pregnancy termination based solely on the NT measurement.

**Figure 1:**
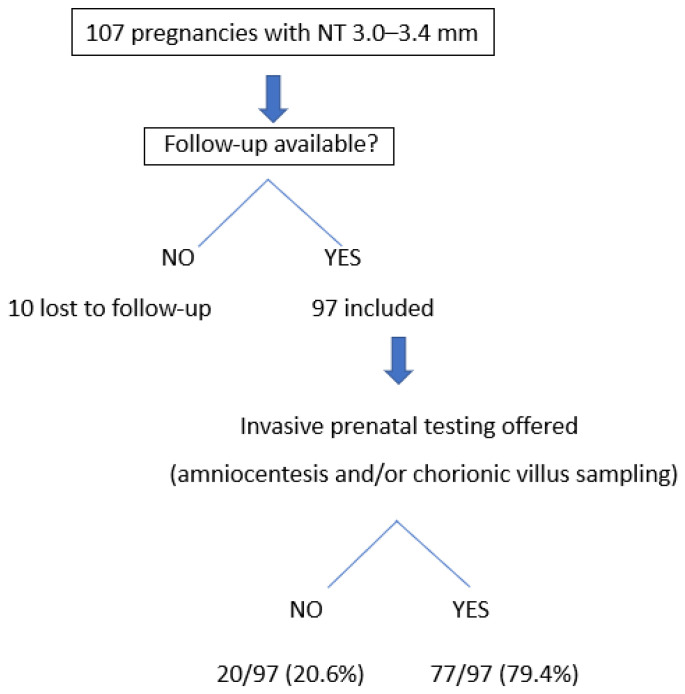
Flowchart of the study population and prenatal diagnostic work-up in pregnancies with NT between 3.0 and 3.4 mm.

Among the 97 patients who continued follow-up, 79.4% (77/97) underwent an invasive diagnostic procedure, while 20.6% (20/97) declined invasive testing (Figure 1).

Within the low-risk group identified by the combined test, one patient out of nine (11%) proceeded with an invasive procedure. Reasons for declining included parental preference to avoid procedure-related risks after counseling, cultural or religious beliefs precluding termination regardless of outcomes, or the decision to rely on NIPT. NIPT was performed elsewhere in 16 of 107 patients (14.9%); among the 11 patients (10.3%) who received low-risk NIPT results, 5 (45%) declined invasive testing, while the other 6 (55%) proceeded with invasive diagnosis, all of which yielded normal genetic results.

Seventy-seven out of 97 women (79.4%) underwent invasive prenatal testing by CVS or amniocentesis.

**Figure 2:**
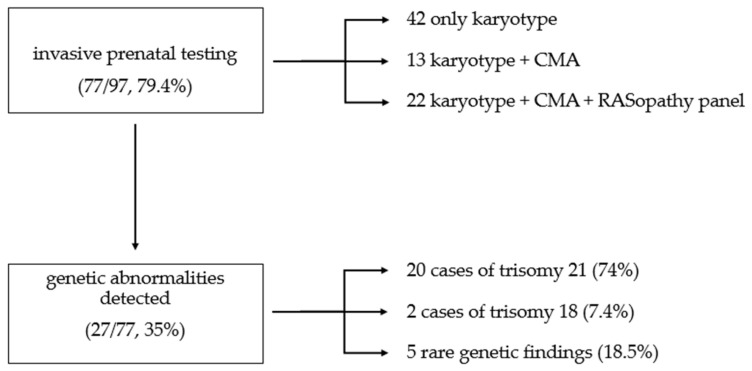
Distribution of invasive prenatal tests performed and genetic abnormalities detected in the study population.

Among these, 42 cases received standard karyotype only, 13 cases received standard karyotype plus CMA, and 22 cases received standard karyotype, CMA and RASopathy gene panel (Figure 2). Genetic abnormalities were detected in 27 of 77 tested fetuses (35%). When considering the entire cohort with complete outcome data, the prevalence of detected genetic abnormalities was 27.8% (27/97). The rate of typical chromosomal abnormalities was 28.5% (22/77), while the rate of rare genetic abnormalities was 6.5% (5/77) (Table 1). All cases of fetal genetic anomalies occurred within the high-risk group.

Trisomy 21 was the most frequent chromosomal aberration, detected in 71.4% of abnormal cases (20/28), followed by two cases of trisomy 18 (7.1%) and two cases of trisomy 22 (7.1%).

Rare findings included two cases detected by CMA: 22q13 de novo microdeletion syndrome and duplication of Xp22.31 (maternally inherited). The RASopathy NGS panel, expanded to include the analysis of the *CHD 7* gene, detected a pathogenic variant in *CHD7* (c.7145dup) that was not inherited from the parents, as well as a VUS of paternal origin in *LZTR.*

In another case, CMA performed on chorionic villus sampling (CVS) identified mosaic duplication of 1q21; however, this finding was not confirmed by subsequent amniocentesis.

The diagnostic yield of CMA, defined as the proportion of additional diagnoses not detectable by standard karyotype, was 5.7% (2/35). The diagnostic yield of RASopathy panel testing was 4.5% (1/22). When considering all cases undergoing advanced genetic testing (CMA and/or RASopathy panel, *n* = 35), the overall diagnostic yield was 8.5% (3/35). When calculated across the entire cohort with complete follow-up, the diagnostic yield was 3% (3/97), as shown in Table 2.

Major ultrasound malformations were detected in 17 of 97 patients (17.5%) (Table 3). Cardiac anomalies were the most frequent structural defects, detected in 9 of the 17 cases (53% of all malformations), corresponding to 8.4% of the entire cohort. The most common cardiac defects were ventricular septal defects, followed by complete atrioventricular canal, tetralogy of Fallot, and transposition of the great arteries.

Of the 17 major fetal anomalies, 10 (59%) were associated with genetic abnormalities detected by karyotyping, chromosomal microarray analysis, or RASopathy panel testing. The remaining cases occurred in fetuses with normal genetic findings.

The rate of termination of pregnancy (TOP) for fetal chromosomal or structural anomalies was 17% (18/107), while the rate of spontaneous miscarriage was 2% (2/107). Of the remaining 72 live-born infants, follow-up was performed until hospital discharge after birth; no systematic postnatal follow-up data beyond this point were available. All available neonatal assessments at discharge were normal and no additional structural anomalies were reported. The demographic and clinical characteristics of the study population are summarized in Table 4 and are expressed as frequencies and percentages.

## 4. Discussion

### 4.1. Interpretation of Principal Findings

In this cohort of 107 pregnancies with NT measurements between 3 and 3.4 mm, we confirmed that this NT range represents a moderate but clinically important risk factor for genetic and structural anomalies.

Although NT values in this range are more common than NT above 3.5 mm [19], their clinical interpretation remains challenging, particularly in the absence of markedly increased combined screening risk [22].

All chromosomal abnormalities in our cohort occurred in pregnancies classified as high-risk at first-trimester combined screening, emphasizing the value of integrating NT measurements with biochemical markers. Only a small proportion of patients with low-risk results at the combined screening decided to undergo invasive testing, with normal results.

### 4.2. Chromosomal Abnormalities in NT Between 3.0 and 3.4 mm

Chromosomal abnormalities are a well-established cause of increased NT. These genetic anomalies account for approximately 30–50% of cases with NT ≥ 3.5 mm, with trisomy 21 being the most frequent [19,21,25,26].

In our cohort, overall, 35% of fetuses that underwent invasive testing were diagnosed with chromosomal anomalies, mainly trisomy 21, followed by trisomy 18 and 22. This distribution is consistent with previous reports in this NT range and reflects differential survival of aneuploidies to the first-trimester screening window [8,9,19].

The chromosomal abnormality rate observed in our study is higher than that reported in several published series focusing on this NT range [8,9,19]. De Vriendt et al. and Petersen et al. [8,9] reported rates between 13% and 17%, while Rybak-Krzyszkowska et al. observed chromosomal aberrations in 18.8% of fetuses with increased combined screening risk [27]. These differences may arise from heterogeneity in local screening, counseling practices and patient selection for invasive testing. Moreover, as a tertiary referral center, we may experience referral bias with enrichment for cases with additional concerning features beyond isolated NT measurements.

### 4.3. Incremental Value of Chromosomal Microarray Analysis

CMA provides a clinically relevant incremental diagnostic yield by detecting submicroscopic CNVs beyond conventional karyotyping. In our cohort, CMA identified pathogenic CNVs in 5.7% of tested cases, a rate consistent with recent multicenter studies reporting yields between 4% and 7% in fetuses with NT measurements between 3.0 and 3.4 mm [8,10,25,26,27,28,29].

Importantly, none of these abnormalities would have been detected by rapid aneuploidy testing or NIPT, underscoring the limitations of targeted screening strategies in this NT range. Although some rare genetic anomalies were detectable by conventional karyotyping, a substantial proportion would have been missed without higher-resolution techniques. These findings support the use of CMA when invasive testing is undertaken, even in fetuses with mildly isolated increased NT at first-trimester assessment. In fetuses with NT measurements between 3 and 4 mm, it should be noted that published diagnostic yield data are often derived from cohorts in which chromosomal analysis was limited to CMA following QF-PCR, without systematic evaluation by conventional karyotyping [9]. In our study, both conventional karyotype and CMA were performed in all cases; therefore, chromosomal abnormalities detectable by karyotyping may have been identified independently of CMA, leading to an apparently lower CMA-specific diagnostic yield compared with previously published series.

### 4.4. Monogenic Disorders and RASopathies

Monogenic disorders, particularly RASopathies, represent another important cause of increased NT in fetuses. Reported detection rates vary widely, with a range between 2.9% and 17.3%, depending on NT measurement and associated findings [30,31,32]. In our cohort, RASopathy panel testing provided an additional diagnostic yield of 4.5%, identifying a pathogenic variant not detectable by cytogenetic methods or CMA.

Although this yield is modest, most published studies focusing on NT values between 3.0 and 3.4 mm have not systematically evaluated monogenic disorders, potentially underestimating their true prevalence. Our results suggest that subtle NT enlargement may be an early phenotypic manifestation of RASopathy-related lymphatic or cardiovascular dysregulation. In this context, and given the current trend toward targeted exome sequencing [22] and broader NGS-based approaches rather than isolated gene panels, our results support the inclusion of RASopathy-related genes—together with other genes implicated in lymphatic and cardiovascular development—within the diagnostic work-up, with expanded prenatal sequencing strategies even for fetuses with NT values between 3.0 and 3.4 mm.

### 4.5. Structural Anomalies and Ultrasound Follow-Up

Structural malformations, particularly congenital heart defects, are strongly associated with increased NT even in the absence of genetic abnormalities [33,34,35,36,37,38,39]. The risk of major cardiac anomalies increases 2.5-fold at NT above the 95th percentile and 10-fold at NT above the 99th percentile [38]. This association reflects shared developmental pathways between cardiac and lymphatic systems, with multiple candidate genes involved in both processes [34]

The 17% rate of structural anomalies in our cohort falls within the range reported [8,9] in similar populations (9–20%). The spectrum of malformations was different, reflecting the heterogeneous etiologies underlying increased NT [33,34,35,36,37,38,39]. However, cardiac defects (predominantly ventricular septal defects and atrioventricular canal defects) were the most common anomalies, representing 53% of structural malformations in our series, consistent with the strong association between increased NT and congenital heart disease extensively reported in the literature [33,34,35,36,37,38,39].

Half of fetuses with major structural anomalies had abnormal genetic findings, while the remainder occurred in fetuses with normal genetic results (Table 3). This underscores the importance of comprehensive ultrasound surveillance regardless of genetic testing outcomes. Early targeted anatomical assessment around 16 weeks, followed by detailed second-trimester evaluation and fetal echocardiography, remains a critical component of prenatal management in this population.

### 4.6. Role and Limitations of NIPT and Evolution of Genetic Testing Strategies

The emergence of NIPT has prompted debate about whether it should replace invasive testing in intermediate-risk scenarios such as NT 3.0–3.4 mm.

Although cfDNA effectively detects common trisomies, it does not eliminate the residual risk of submicroscopic chromosomal abnormalities, monogenic disorders, or structural malformations. Petersen et al. calculated that after normal NIPT for common trisomies, the residual risk for submicroscopic aberrations in NT 3.0–3.4 mm is 4.8% (1:21) [2]. Rybak-Krzyszkowska et al. found that cell-free DNA testing (cfDNA) would miss 1.8% (1:54 risk) of clinically significant abnormalities in this NT range [27].

In our cohort, NIPT would have missed all rare genetic findings and could not address the substantial risk of major fetal anomalies. Notably, a significant proportion of patients with low-risk NIPT results still elected to undergo invasive testing, reflecting persistent parental concern about residual risk. These findings, together with the existing literature [2,8,9,13,25], support the recommendation that cfDNA should not replace invasive diagnostic testing in pregnancies with borderline increased NT.

Over the nearly nine-year study period, genetic testing strategies in our center evolved substantially. With progressive incorporation of CMA and, more recently, driven by emerging evidence on the association between borderline NT elevations and early lymphatic or cardiovascular dysfunction, our institution implemented a revised internal protocol extending RASopathy testing to pregnancies with NT ≥ 3.0 mm.

This temporal heterogeneity may have influenced detection rates, particularly for monogenic disorders, and should be considered when interpreting our results.

Overall, this evolution in diagnostic practice highlights the dynamic nature of prenatal genetics and underscores the importance of continuously adapting clinical protocols to incorporate new evidence and emerging technologies.

### 4.7. Strengths and Limitations

Our study has several strengths. The systematic identification of all cases meeting the inclusion criteria minimizes selection bias. The comprehensive phenotyping with detailed ultrasound surveillance and multi-modal genetic testing (karyotype, CMA, RASopathy panels) provides more complete characterization than studies relying on karyotype alone.

The long study period provides sufficient sample size to detect clinically meaningful rates of rare outcomes while encompassing the evolution of genetic testing technologies from conventional karyotyping through CMA to targeted gene panels. The single-center design ensures consistency in measurement techniques, counseling approaches, and follow-up protocols throughout the study period. All NT measurements were performed by certified sonographers using standardized techniques, and all genetic testing was performed in accredited laboratories, enhancing the reliability of results.

Limitations include the retrospective design, referral bias inherent to a tertiary center, and selective application of advanced genetic testing reflecting evolving institutional protocols. Invasive testing was not performed in all cases, potentially leading to selection bias and overestimation of the diagnostic yield. However, this reflects real-world clinical practice, where the decision to undergo invasive testing is influenced by parental choice and additional ultrasound findings. Nevertheless, these factors affect the magnitude of risk estimates rather than the fundamental conclusion that NT 3.0–3.4 mm represents a high-risk category warranting comprehensive evaluation.

### 4.8. Clinical Implications and Recommendations

Several important research questions remain unanswered. First, the optimal genetic testing strategy for NT 3.0–3.4 mm requires further clarification. Should chromosomal microarray be performed universally or only after normal karyotype? Should RASopathy testing be performed universally, selectively based on phenotype, or only after normal karyotype and CMA? The cost-effectiveness of different testing algorithms needs formal evaluation.

Second, the role of emerging technologies including exome sequencing and genome sequencing in prenatal diagnosis of increased NT cases requires investigation. Several studies have demonstrated incremental yield from exome sequencing in structurally abnormal fetuses with normal microarray [22]. Whether this yield justifies the additional cost, lengthy turnaround time, and high rate of VUS in prenatal settings remains debated.

Third, long-term neurodevelopmental outcomes in children born after prenatal diagnosis of isolated increased NT with normal genetic testing deserve systematic study. Some evidence suggests increased risks of subtle neurodevelopmental delays even in the absence of major structural abnormalities [3], but data specifically addressing the NT 3.0–3.4 mm range are limited.

Fourth, the psychological impact of prenatal diagnosis in intermediate-risk situations requires attention. Parents facing uncertain prognoses or VUS experience significant anxiety and decisional conflict. Research on optimal counseling strategies and decision-support tools could improve patient experience and decision quality.

Based on our findings and the contemporary literature, we propose the following clinical recommendations for management of fetuses with NT 3.0–3.4 mm:

Genetic Counseling: All patients with NT 3.0–3.4 mm should receive genetic counseling regardless of combined screening risk calculation, maternal age, or family history. Counseling should address the spectrum of possible etiologies including chromosomal abnormalities, submicroscopic copy-number variants, single-gene disorders including RASopathies, and structural malformations with or without genetic abnormalities.

Invasive Testing: Invasive prenatal diagnosis should be offered to all patients with NT 3.0–3.4 mm. The choice between chorionic villus sampling and amniocentesis should be based on gestational age, urgency of results, maternal preference, and technical feasibility. Patients declining invasive testing should be counseled about the substantial residual risks and limitations of alternative approaches, including cell-free DNA screening.

Genetic Testing Protocol: Invasive samples should undergo chromosomal microarray analysis as the first-line test rather than conventional karyotype alone, given the 5–6% incremental yield of microarray and superior detection of clinically relevant copy-number variants [10,11,12,13].

Rapid aneuploidy detection by QF-PCR can provide preliminary results within 24–48 h while awaiting definitive microarray results.

RASopathy Testing: RASopathy testing should be considered when NT is between 3 and 3.4 mm.

Ultrasound Surveillance: Comprehensive anatomical survey should be performed at 16–18 weeks, with a detailed morphology scan at 20–22 weeks. Fetal echocardiography should be performed in all cases, given the 53% cardiac malformation rate, regardless of genetic testing results.

Multidisciplinary Approach: Complex cases with multiple abnormalities, variants of uncertain significance, or discordant findings between genetic and ultrasound assessments should be reviewed by multidisciplinary teams, including maternal–fetal medicine specialists, clinical geneticists, pediatric cardiologists, and neonatologists to provide comprehensive counseling and optimize management planning.

### 4.9. Ethical and Medico-Legal Considerations

The identification of NT between 3 and 3.4 mm requires accurate counseling and careful documentation of all proposed diagnostic options. Pregnant women and their partners must receive clear and balanced information regarding the potential genetic and structural risks associated with NT 3.0–3.4 mm, as well as the limitations of first-trimester screening and non-invasive tests.

Accurate documentation of counseling sessions, patient decisions, and follow-up plans is essential both for ethical practice and medico-legal protection. Adherence to standardized guidelines and institutional protocols ensures consistent risk assessment and demonstrates professional diligence. When patients decline recommended follow-up or invasive testing, the clinician’s responsibility lies in providing complete information and appropriate recommendations; however, autonomous patient decisions cannot be held as clinician errors.

Failure to follow these principles may increase medico-legal exposure, particularly if significant anomalies are subsequently detected.

Current international guidelines [1] and recommendations do not universally recommend chromosomal microarray analysis (CMA) in fetuses with NT between 3.0 and 3.4 mm when no structural anomalies are present [39]. However, our findings demonstrate that clinically significant abnormalities—including mosaicisms, structural chromosomal rearrangements, and copy-number variants—may occur in this intermediate NT range. Importantly, our results reinforce that neither cfDNA nor QF-PCR is sufficient in this subgroup, and that full conventional cytogenetic karyotyping should be considered the baseline genetic investigation when invasive testing is performed. This strengthens the argument for re-evaluating current guideline thresholds and supports more nuanced, risk-adapted prenatal management.

In summary, the management of NT in the range 3.0–3.4 mm requires a structured approach that integrates technical accuracy, guideline-based practice, transparent communication, and traceable decision-making, thereby safeguarding patient autonomy and minimizing medico-legal risks [40,41,42,43,44,45].

## 5. Conclusions

In fetuses with mildly increased nuchal translucency (NT 3.0–3.4 mm), advanced genetic testing provides clinically relevant additional information beyond standard screening. None of the identified rare genetic abnormalities would have been detected by QF-PCR or NIPT, and standard karyotype alone would have missed several clinically significant findings.

Full conventional karyotyping should remain the minimum diagnostic test, not only to detect aneuploidies but also to identify structural rearrangements. Approximately 10% of trisomy 21 cases result from translocations, often inherited from an unaffected parent, with important implications for recurrence risk and guidance for future testing, including uniparental disomy (UPD) analysis. CMA contributed an additional 5.7% diagnostic yield, while the RASopathy panel added 4.5%, identifying pathogenic variants not detectable by cytogenetic methods.

When CMA is offered as the first-tier test, karyotyping should be considered after CMA if results suggest the presence of balanced rearrangements, or when clinical suspicion remains despite a normal CMA result, in order to detect chromosomal abnormalities not identified by array-based techniques.

Trio analysis (testing both parents and the fetus) can strengthen variant interpretation and accelerate prenatal decision-making, allowing distinction between de novo and inherited variants and informing recurrence risk counseling.

We recommend a comprehensive, risk-adapted prenatal strategy that includes karyotyping, CMA, and selected gene panels, combined with detailed sequential ultrasound assessment including fetal echocardiography. This approach improves diagnostic accuracy, allows early detection of evolving structural malformations, and informs parental counseling in this intermediate-risk population.

## Figures and Tables

**Table 1:** Rare genetic findings identified by G-banded karyotype, CMA and RASopathy NGS panel in cases with NT between 3.0 and 3.4 mm.

Rare Genetic Finding	NT (mm)	Genetic Diagnostic Method	Associated Fetal Structural Anomalies	Pregnancy Outcome
Xp22.31 duplication involving genes *HDHD1*, *STS*, *VCX*, and *PNPLA4* inherited from mother	3.20	CMA	Persistent nuchal edema	Livebirth
Trisomy 22	3.12	Full cytogenetic karyotype	No abnormalities detected at 11–13 weeks	TOP at 14 weeks
Trisomy 22	3.19	Full cytogenetic karyotype	TOF and IUGR at 16 weeks	TOP at 18 weeks
22q13 deletion syndrome (de novo)	3.37	CMA	VSD at 16 weeks	TOP at 17 weeks
Pathogenic variant in the *CHD7* gene (de novo) + VUS in *LZTR1* gene	3.12	RASopathy NGS panel	Not evidence of fetal anomalies in the first and second trimester	TOP at 22 weeks

CMA: chromosomal microarray analysis, TOP: termination of pregnancy, IUGR: intrauterine growth restriction, TOF: tetralogy of Fallot, VSD: ventricular septal defect.

**Table 2:** Diagnostic yield of genetic testing in fetuses with NT 3.0–3.4 mm: pathogenic findings not detectable by standard karyotype.

Test	Number of Tests Performed	Pathogenic Findings (*n*)	Diagnostic Yield (%)
CMA (incremental)	35	2	5.7
RASopathy NGS panel	22	1	4.5
Advanced genetic testing (overall) *	35	3	8.5
Entire cohort with follow-up	97	3	3

* Advanced genetic testing includes CMA and/or RASopathy NGS panel.

**Table 3:** Fetal congenital defects and related genetic findings in cases with increased nuchal translucency (NT between 3.0 and 3.4 mm).

Case	NT (mm)	Type of Procedure for Genetic Analysis	Structural Anomaly	Gestational Age at Diagnosis of Fetal Defect	Genetic Findings	Pregnancy Outcomes
1	3.00	CVS	LCDH	20 weeks	Normal karyotype and CMA	Livebirth
2	3.44	Amniocentesis	IUGR	16 weeks	Trisomy 21	TOP
3	3.44	CVS	Cystic hygroma	13 weeks	Trisomy 21	TOP
4	3.39	CVS	SUA	20 weeks	Normal karyotype and CMA	Livebirth
5	3.35	CVS	AVSD	12 weeks	Normal karyotype and CMA	TOP
6	3.00	Amniocentesis	VSD, mild bilateral pelvic dilatation, short long bones	16 weeks	Trisomy 21	TOP
7	3.20	CVS	Absent nasal bone, AVSD	12 weeks	Trisomy 21	TOP
8	3.40	Amniocentesis	IUGR, choroid plexus cysts, dilated gallbladder	15 weeks	Trisomy 18	TOP
9	3.19	CVS	TOF	16 weeks	Trisomy 22	TOP
10	3.10	CVS	Bilateral clubfoot, persistent nuchal edema	20 weeks	Normal karyotype	Livebirth
11	3.44	CVS	Omphalocele	12 weeks	Trisomy 21	TOP
12	3.42	CVS	TOF	17 weeks	Normal karyotype and CMA, normal RASopathy	TOP
13	3.37	CVS	VSD	16 weeks	22q13 deletion syndrome	TOP
14	3.43	CVS	VSD	20 weeks	Normal karyotype and CMA	Livebirth
15	3.10	CVS	Outflow tract abnormality	13 weeks	Trisomy 21	TOP
16	3.10	Amniocentesis	Short long bones	30 weeks	Normal karyotype and CMA, normal RASopathy	Livebirth
17	3.43	Amniocentesis	Tricuspid regurgitation	20 weeks	Normal karyotype and CMA, normal RASopathy	Livebirth

LCDH: left congenital diaphragmatic hernia, SUA: single umbilical artery, CVS: chorionic villus sampling, IUGR: intrauterine growth restriction, TOF: tetralogy of Fallot, TOP: termination of pregnancy, AVSD: atrioventricular septal defect, VSD: ventricular septal defect.

**Table 4:** Demographic characteristics of the study population.

Maternal Characteristics	*n* = 97
Age, years	35.3 (±5.6)
Geographic origin Caucasian *n* (%) Black African *n* (%) South Asian *n* (%) East Asian *n* (%)	90 (92.7) 2 (2.06) 3 (3.09) 2 (2.06)
Height, cm	165 (±6.1)
Weight, kg	63.3 (±12.59)
BMI	20.9 (±4.4)
Smoking, yes	12 (12.3)
Method of conception Natural (%) Assisted by ovulation drugs/IUI (%) IVF (%)	85 (87.6) 2 (2.06) 10 (10.3)
Nulliparous, yes	35 (36)
Pregestational diabetes	1 (1.03)
CHT	1 (1.03)
APS	2 (2.06)

Note: Data are presented as number (percentage) or mean (±standard deviation). CHT—chronic hypertension; IVF—in vitro fertilization; BMI—body mass index; APS—antiphospholipid syndrome.

## Data Availability

The data presented in this study are available on request from the corresponding author due to the privacy policies of the hospitals involved.
